# Supplementary material for: Growth under cold conditions in a wide perennial ryegrass panel is under tight physiological control
Source: PeerJ. 2018 Sep 11;6:e5520. doi: 10.7717/peerj.5520 (PMC6138037; doi:10.7717/peerj.5520)
Supplement: Table S1 [file peerj-06-5520-s001.docx]

**Supplementary Materials Table 1**: The fifty-seven accessions of perennial ryegrass, their origin, ploidy status, maturity group and further information:

| Accession | Breeder | Ploidy | Maturity Group | Recommended List |
| --- | --- | --- | --- | --- |
| AberAvon | IBERS | 2n | Late |  |
| AberChoice | IBERS | 2n | Late | Ireland |
| AberClyde | IBERS | 4n | Intermediate |  |
| AberDart | IBERS | 2n | Intermediate |  |
| AberGain | IBERS | 4n | Late | Ireland |
| AberGreen | IBERS | 2n | Intermediate |  |
| AberZeus | IBERS | 2n | Intermediate |  |
| Arara | Agroscope | 2n |  | Switzerland |
| Arolus | Agroscope | 2n |  | Switzerland |
| Astonenergy | EuroGrass | 4n | Late | Ireland |
| Ba 14088 | IBERS | 2n | Late |  |
| Ba 14125 | IBERS | 2n | Late |  |
| Ba 14155 | IBERS | 2n | Intermediate |  |
| BAR 01 | Barenbrug | 2n |  | Romania |
| BAR 02 | Barenbrug | 2n |  | Romania |
| BAR 03 | Barenbrug | 2n |  | Romania |
| BAR 04 | Barenbrug | 2n |  | Romania |
| BAR 05 | Barenbrug | 2n |  | Netherlands |
| BAR 06 | Barenbrug | 2n |  | Netherlands |
| BAR 07 | Barenbrug | 2n |  | Netherlands |
| BAR 08 | Barenbrug | 2n |  | France |
| BAR 09 | Barenbrug | 2n |  | New Zealand |
| BAR 10 | Barenbrug | 2n |  | New Zealand |
| Cancan | Limagrain / DLF | 2n | Late | Ireland |
| Carraig | Teagasc | 4n | Intermediate | Ireland |
| Cashel | Teagasc | 2n | Intermediate | Ireland |
| Denver | Advanta / DLF | 2n | Late | Ireland |
| Giant | Teagasc | 4n | Intermediate | Ireland |
| Glencar | Teagasc | 4n | Late | Ireland |
| Glenveagh | Teagasc | 2n | Late | Ireland |
| January | Teagasc | 2n | Early | Ireland |
| Kintyre | Teagasc | 4n | Late | Ireland |
| LP0035 | Agroscope | 2n |  | Based on highland ecotypes |
| LP0055 (AROTIS) | Agroscope | 2n |  | Switzerland |
| LP0075 | Agroscope | 2n |  |  |
| LP0105 | Agroscope | 2n |  | Based on highland ecotypes |
| LP0425 | Agroscope | 2n |  | Based on highland ecotypes |
| LP0515 | Agroscope | 2n |  | Based on highland x low land ecotypes |
| LP1005 | Agroscope | 2n |  | Based on highland x low land ecotypes |
| LP9155 (CANIS) | Agroscope | 2n |  | Released for German market |
| LP9435 (ARUGA) | Agroscope | 2n |  | Switzerland |
| LP9535 | Agroscope | 2n |  |  |
| Magician | Teagasc | 4n | Intermediate | Ireland |
| Majestic | Teagasc | 2n | Late | Ireland |
| Millennium | Teagasc | 4n | Late | Ireland |
| Picadilly | EuroGrass | 2n | Late | Ireland |
| Premium | Innoseeds /DLF | 2n | Intermediate | Ireland |
| RHZ110123 Ottoberg | Swiss ecotypes | 2n |  | Low land ecotype |
| RHZ110124 Mümliswil Passwang | Swiss ecotypes | 2n |  | High land ecotype |
| RHZ110125 Wildberg | Swiss ecotypes | 2n |  | Low land ecotype |
| RHZ110127 Bütschwil Zwiselen | Swiss ecotypes | 2n |  | Low land ecotype |
| Rodrigo | EuroGrass | 2n | Intermediate | Ireland |
| Rosetta | AFBI | 2n | Intermediate | Ireland |
| Solomon | Teagasc | 2n | Intermediate | Ireland |
| Soriento | EuroGrass | 2n | Late | Ireland |
| Stefani | DLF | 2n | Late | Ireland |
| Twymax | CPB Twy. /DLF | 4n | Late | Ireland |
