# Supplementary material for: Growth under cold conditions in a wide perennial ryegrass panel is under tight physiological control
Source: PeerJ. 2018 Sep 11;6:e5520. doi: 10.7717/peerj.5520 (PMC6138037; doi:10.7717/peerj.5520)
Supplement: Table S2 — Irish weather data averages from Met Éireann (The Irish Meterological Service, www.met.ie ) for January, February, March, April and May of the weather stations and years: Malin Head 1981–2010, Kilkenny 1978–2007, Shannon Airport 1981–2010, Dublin Airport 1981–2010, Birr 1979–2008, Belmullet 1981–2010, Clones 1978–2007, Cork Airport 1981–2010, Mullingar 1979–2008, Rosslare 1978-2007 and Valentia 1981–2010. [file peerj-06-5520-s002.docx]

**Supplemental Materials Table** **2** Irish weather data averages from Met Éireann (The Irish Meterological Service, [www.met.ie](http://www.met.ie)) for January, February, March, April and May of the weather stations and years: Malin Head 1981–2010, Kilkenny 1978–2007, Shannon Airport 1981–2010, Dublin Airport 1981–2010, Birr 1979-2008, Belmullet 1981–2010, Clones 1978–2007, Cork Airport 1981–2010, Mullingar 1979–2008, Rosslare 1978-2007 and Valentia 1981–2010

|  | January | February | March | April | May |
| --- | --- | --- | --- | --- | --- |
| Average daily maximum in °C | 8.33 | 8.56 | 10.20 | 12.15 | 14.78 |
| Average daily minimum in °C | 2.86 | 2.85 | 3.98 | 5.11 | 7.37 |
| Average daily temperature in °C | 5.62 | 5.71 | 7.10 | 8.62 | 11.07 |
| Average relative humidity in % at 0900UTC | 87.41 | 86.77 | 85.11 | 80.75 | 78.16 |
| Average relative humidity in % at 1500UTC | 81.45 | 76.86 | 73.72 | 69.65 | 69.36 |
