# Supplementary material for: Growth under cold conditions in a wide perennial ryegrass panel is under tight physiological control
Source: PeerJ. 2018 Sep 11;6:e5520. doi: 10.7717/peerj.5520 (PMC6138037; doi:10.7717/peerj.5520)
Supplement: Figure S2 — The letters represent significant different groups according to an ANOVA test and a Tukey ranking test. Both accession and treatment were highly significant. [file peerj-06-5520-s004.pdf]

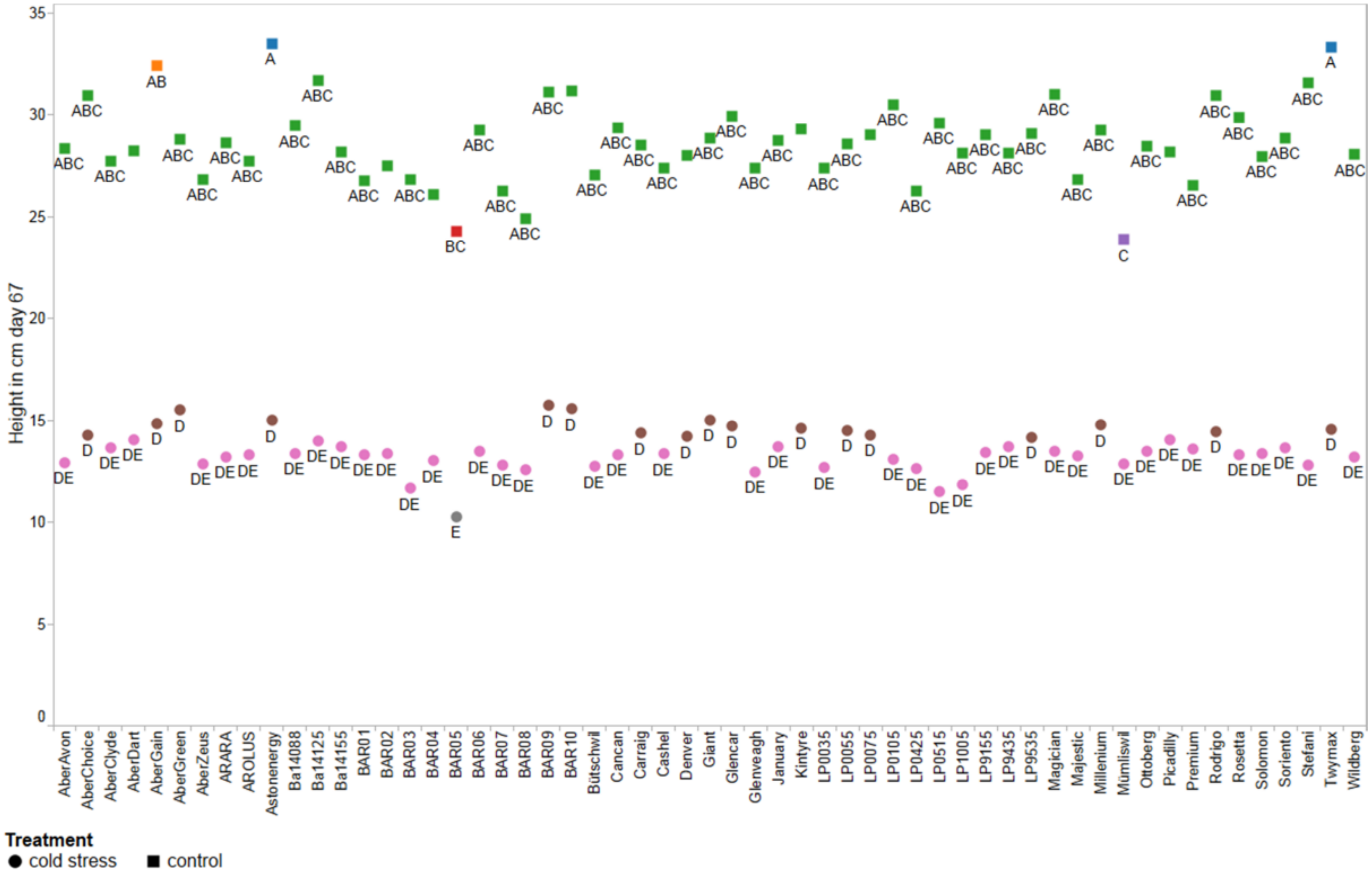

Supplemental Materials Figure 2: Plant height (cm) at day 67 for the 57 accessions grown under cold stress and control conditions. The letters represent significant different groups according to an ANOVA test and a Tukey ranking test. Both accession and treatment were highly significant.
