# Supplementary material for: Growth under cold conditions in a wide perennial ryegrass panel is under tight physiological control
Source: PeerJ. 2018 Sep 11;6:e5520. doi: 10.7717/peerj.5520 (PMC6138037; doi:10.7717/peerj.5520)
Supplement: Figure S4 — The letters represent significant different groups according to an ANOVA test and a Tukey ranking test. Neither factor, accession or treatment, was significant. [file peerj-06-5520-s006.pdf]

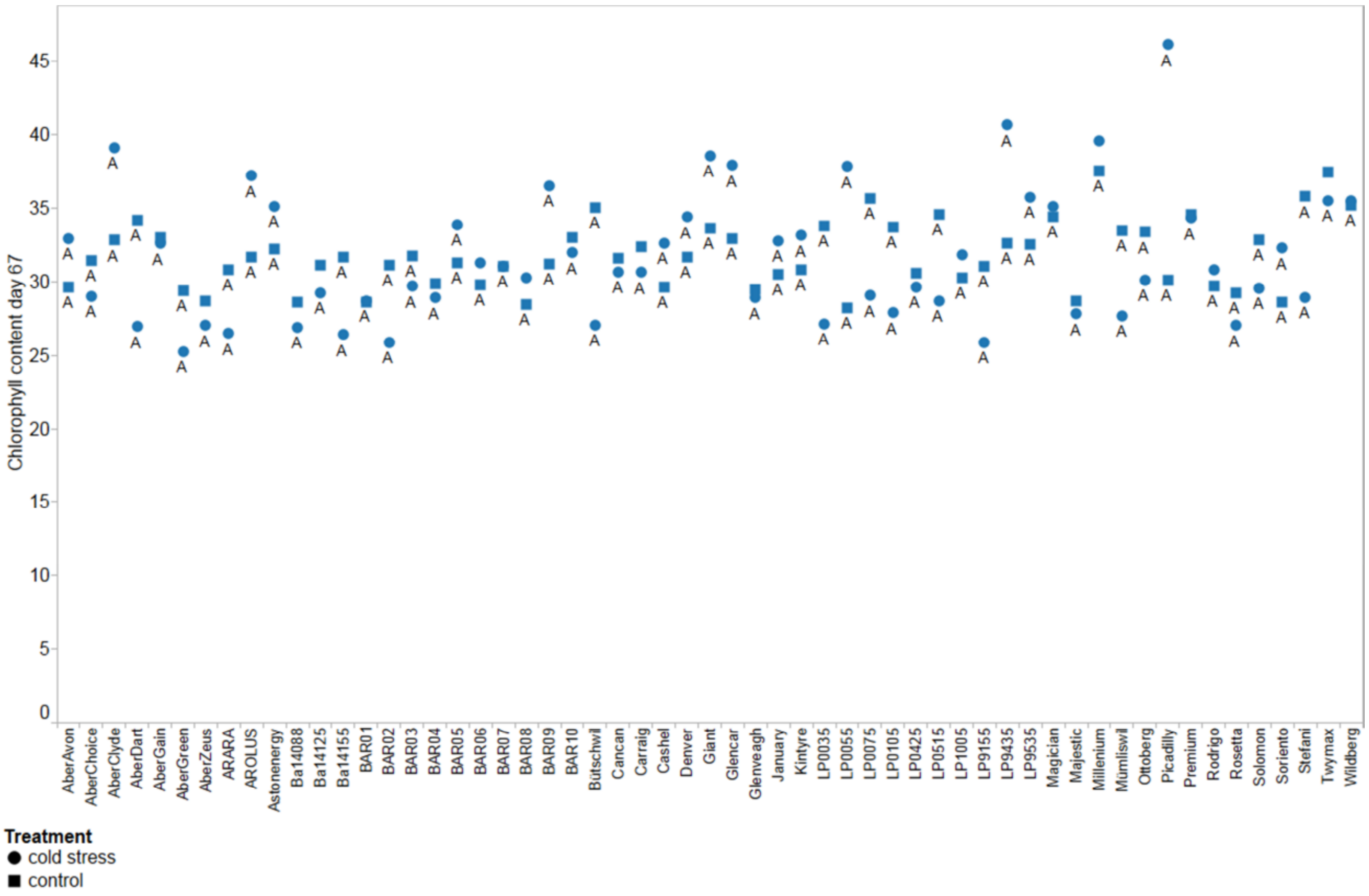

Supplemental Materials Figure 4: Chlorophyll content at day 67 of the 57 accessions grown under cold stress and control conditions. The letters represent significant different groups according to an ANOVA test and a Tukey ranking test. Neither factor, accession or treatment, was significant.
