# Supplementary material for: Growth under cold conditions in a wide perennial ryegrass panel is under tight physiological control
Source: PeerJ. 2018 Sep 11;6:e5520. doi: 10.7717/peerj.5520 (PMC6138037; doi:10.7717/peerj.5520)
Supplement: Figure S5 [file peerj-06-5520-s007.pdf]

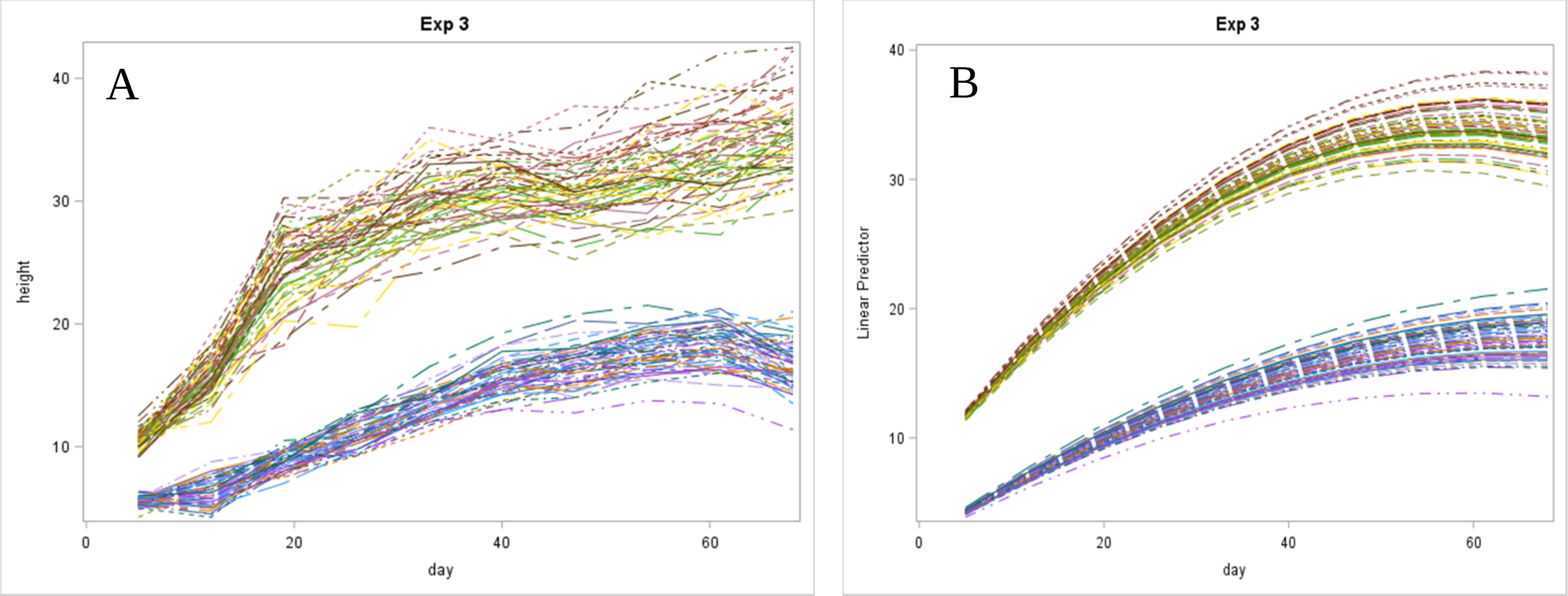

Supplemental Materials Figure 5: (A) Plotted growth height raw data of experiment 3 for control and cold treatment and (B) modelled growth height data of experiment 3 for control and cold treatment.
